# Exploration of rehabilitation psychology in multidisciplinary care of upper extremity peripheral nerve injuries: patient and provider perspectives

**DOI:** 10.3389/fresc.2026.1742388

**Published:** 2026-05-01

**Authors:** Stephanie H. Vu, Michael L. Dolezal, Sonja Katt, JulieAnn Uh, Benjamin L. Grannan, Sarah M. Smith, Christopher S. Crowe, Yusha Katie Liu, Jeffrey B. Friedrich

**Affiliations:** 1 Division of Plastic Surgery, Department of Surgery, University of Washington, Seattle, WA, United States; 2 George Washington University School of Medicine and Health Sciences, Washington, DC, United States; 3 Division of Rehabilitation Psychology and Neuropsychology, Department of Rehabilitation Medicine, University of Washington School of Medicine, Seattle, WA, United States; 4 Department of Neurological Surgery, University of Washington, Seattle, WA, United States; 5 Department of Rehabilitation Medicine, University of Washington, Seattle, WA, United States

**Keywords:** brachial plexus neuropathies, multidisciplinary care team, nerve compression, peripheral nerve injuries, rehabilitation psychology

## Abstract

Peripheral nerve injuries pose long-term functional and psychological challenges, necessitating a multidisciplinary approach that integrates surgical, medical, and rehabilitative care. Rehabilitation psychologists play a critical role in addressing emotional distress and functional adaptation after injury, particularly as treatment decisions shape recovery expectations. This study examines how factors such as injury chronicity, mechanism, and surgical decisions impact patients’ perception of the value of rehabilitation psychology (RP) intervention following peripheral nerve injury. RP intervention consisted of seeing a rehabilitation psychologist for mental health screening, as well as recommendations for further mental health related follow-up. Fifty consecutive patients with upper extremity peripheral nerve injuries who underwent consultation at a multidisciplinary peripheral nerve clinic completed a two-item survey to assess the perceived importance of RP before and after their clinic visit. Providers were asked to complete a five-question survey. Patients were grouped based on their perception of the value of following RP intervention, and predictors of improved perception were analyzed. Following intervention, 74% of patients demonstrated improved perception of RP or stayed at the highest possible score both pre- and post-visit, suggesting that RP was found to be a valuable addition to the PNI care team. No statistically significant associations were found between perception changes and clinical factors. These findings support the integration of RP in multidisciplinary peripheral nerve injury care, and further research should explore the long-term role of RP and applicability to other injury types.

## 1 Introduction

Peripheral nerve injuries (PNIs) are life-altering conditions that can lead to persistent sensory and motor deficits, chronic neuropathic pain, and significant reductions in quality of life (1–5). In addition to the physical impairments, PNIs can often significantly impact one's psychological well-being and social function (6–8). The pain and functional changes associated with these injuries can present considerable coping challenges, with many individuals reporting symptoms of psychological distress, such as depression, anxiety, and posttraumatic stress, especially when following traumatic etiologies (9, 10). Even more, these symptoms may be further exacerbated by factors like pain catastrophizing and can contribute to greater functional impairment than would be expected based on physical injury alone (11–13). While numerous advances in surgical techniques and rehabilitation strategies have improved outcomes for physical function, there remains a critical need to address the psychological dimensions of recovery, which are often underrecognized and undertreated in routine care.

Despite its well-established impact on the overall health and functioning of individuals with PNI, psychological support is rarely integrated into the current standard clinical management. Evidence from other surgical populations demonstrates early psychological intervention, including cognitive behavioral therapy, motivational interviewing, and acceptance and commitment therapy, can reduce pain, anxiety, and disability while improving recovery and quality of life in cases involving significant loss of function (14–24). Effective management of PNIs increasingly calls for a multidisciplinary approach that integrates surgical care, physical rehabilitation, and psychosocial support. Yet, rehabilitation psychology (RP) remains a promising yet underutilized component in this framework (9, 25). To date, the potential role and need for RP intervention in the context of PNIs deserves further exploration.

In this context, we integrated RP as core members of our multidisciplinary peripheral nerve clinic care team. Patients are scheduled to meet with a rehabilitation psychologist during the same clinic visit and in the same physical space as the surgical and rehabilitation teams, most often at the time of their initial new patient consultation. The sequencing of provider visits is determined collaboratively by the care team and informed by the surgical plan and injury severity, with RP consultations occurring either before or after the surgical evaluation depending on what is expected to be most clinically beneficial. Our RP providers are licensed rehabilitation psychologists assigned to the peripheral nerve clinic due to their specialized expertise in the psychological aspect of rehabilitation. By embedding RP into the clinic's standardized care pathway, psychological care is timely, coordinated with injury rehabilitation goals, and positioned as a routine and accessible element of care. This collaborative model ensures that psychological assessment and intervention are aligned with both surgical decision-making and nonsurgical rehabilitation goals. Therefore, this exploratory study aims to assess the clinical utility of RP within a multidisciplinary care model by examining both provider and patient perspectives on perceived benefit and ongoing need. These insights can guide future program development and inform broader implementation of psychological support in multidisciplinary peripheral nerve injury care clinics.

## 2 Methods

### 2.1 Participants and procedure

Fifty consecutive patients with upper extremity PNIs who presented for consultation in the multidisciplinary peripheral nerve clinic were included in this cross-sectional clinic-based evaluation. Inclusion criteria included age ≥18 and attendance at the multidisciplinary peripheral nerve clinic. Patients who were incarcerated, pregnant, or seen by a rehabilitation psychologist not assigned to the multidisciplinary peripheral nerve clinic were excluded. There were no additional exclusion criteria with the intent of reducing selection bias. Data were retrospectively collected on demographic information, injury mechanism, and treatment pathways, including both surgical and non-surgical methods. The study procedures were performed according to the approved University of Washington Institutional Review Board (IRB) protocol.

Participants were recruited from a multidisciplinary peripheral nerve clinic located in the Western United States over a four-month period in 2024. Given that the goal of this study was an exploratory analysis of the need of RP, we aimed to enroll a sample size of 50 patients. Patients presented to the clinic for diagnosis and evaluation by a multidisciplinary team, which includes a rehabilitation psychologist. As part of patients’ initial visit, they met the rehabilitation psychologist to evaluate their current psychological distress and coping related to their injury and associated pain and functional limitations. This was done using a brief, bespoke semi-structured interview that assessed current functioning, pain, sleep, symptoms of common psychological disorders (depression, anxiety, posttraumatic stress disorder), and substance use. At the end of the interview, the rehabilitation psychologist provided brief intervention as needed and recommended follow-up with RP or other services if indicated based on their clinical opinion.

Members of the research team conducted a preliminary chart review to assess eligibility for each patient presenting to the multidisciplinary nerve clinic for initial evaluation. The research team then approached eligible patients for study recruitment after the patient received care with the rehabilitation psychologist and medical care team.

### 2.2 Survey instruments

Participants who consented to the study completed a two-item survey assessing the patients’ perception of the need of RP, consisting of the following questions:

1. Before your visit, how important did you believe it was to meet with the Rehabilitation Psychologist during your visit to the nerve clinic today? (0–10 scale, 0 = not useful, 10 = extremely useful)
2. After seeing the Rehabilitation Psychologist, how valuable did you believe it was for you to meet with the Rehabilitation Psychologist today? (0–10 scale, 0 = not useful, 10 = extremely useful)

For each patient, the rehabilitation psychologist completed a five-item survey assessing the common clinical indications for RP services and outlined ongoing recommendations for each patient seen in the clinic, consisting of the following questions:

1. What is your assessment of the value of rehabilitation psychology services provided to patients in the Harborview Medical Center (HMC) nerve clinic?
2. What are the most common reasons/indications for the services you have provided to HMC nerve clinic patients?
3. What is your estimate about frequency of patients who need ongoing rehabilitation psychology care? (%)
4. What type of services are you typically recommending for ongoing treatment for patients?
5. What aspects of the nerve clinic can be improved specifically with respect to provision of rehabilitation psychology services?

Though these questions ask about clinic-level perceptions, providers were asked to answer these in reference to each patient. This provider questionnaire was designed to capture the perceived value of RP services (question 1) and the most common indications and services provided (questions 2). Themes related to provider recommendations and session topics from question 2 were identified through manual review of the survey answers by the research team and rehabilitation psychologists, and the frequency of themes mentioned were calculated according to how often each theme appeared across responses, with some patients assigned more than one theme. The following three questions assessed the frequency of ongoing care recommendations (question 3), recommended services for continued care (question 4), and patient-specific suggestions for improving RP services (question 5); however, data from these latter three questions were not used for the purposes of this present study.

### 2.3 Data preparation and analysis

To prepare data for analysis, we used abstracted data on handedness (left, right, ambidextrous) and injury laterality (left, right, both) to classify patients according to whether their dominant hand was affected by the PNI. Patients were considered to have dominant hand affected when handedness and injury laterality corresponded. Ambidextrous patients with a unilateral injury were classified as having their dominant hand unaffected by PNI, as they retained functional use of the contralateral equally dominant hand. We also evaluated the responses from the rehabilitation psychologist providers’ survey question 2 and categorized them into the following themes: Social Support, Recourse Connection/Coordination, Safety Planning and PTSD Support, Substance Use Assessment, Values/Goals Review, Mood Management, Mental Health Support, Sleep Management, Coping and Resiliency Support, Pain Management, Adjustment to Injury, and Not Helpful.

Prior to analysis, we evaluated whether statistical assumptions were violated for each statistical test. The assumption of normality was considered violated if skew and kurtosis were greater than the absolute values of 3 and 10, respectively (26) and if the Shapiro–Wilk test was statistically significant. For the independent samples t-tests and analysis of variance (ANOVA), Levene's test was used to evaluate the assumption of homogeneity of variance, with a statistically significant result indicating that this assumption was violated. Visual inspection of the histogram also suggested non-normality, so we elected to proceed with nonparametric tests.

The first exploratory analyses investigated patients’ perceptions of the benefit of RP services in the multidisciplinary clinic setting. Descriptive statistics were used to summarize patients’ perceived value of meeting with the rehabilitation psychologist before and after their clinic visit, as well as the change in perceived value. Change scores were calculated by subtracting each patient's pre-visit rating from their post-visit rating. A paired samples *t*-test was conducted to determine whether patients’ perceptions significantly changed from before to after meeting with the rehabilitation psychologist. Independent samples *t*-tests were used to explore whether patients’ change scores differed based on gender, operative status, and whether the patient's dominant hand was affected by the PNI. One-way ANOVA were conducted to explore whether patients’ change scores differed based on injury etiology, and a simple linear regression was used to assess the relationship between age and change scores. Effect sizes were calculated using Cohen's *d* for all *t*-tests (*d* = 0.20–0.50 considered as a small effect, *d* = 0.50–0.80 as medium, and *d* ≥ 0.80 as large) (27) and *η*^2^ for the one-way ANOVA (*η*2 = 0.04 considered as a small effect, *η*^2^ = 0.25 medium, and *η*^2^ = 0.64 large).

The next exploratory analyses investigated providers’ perceptions of which patients would benefit from RP services in the multidisciplinary clinic setting, as well as how these perceptions were associated with demographic and injury-related characteristics. Descriptive statistics were used to summarize the frequency that the rehabilitation psychologists recommended follow-up for psychology services. Chi-square (*χ*^2^) tests of independence were conducted to explore associations between providers’ follow-up recommendations (yes/no) and the following categorical demographic and injury-related characteristics: gender, injury mechanism, operative status, and whether the patient's dominant hand was affected by the PNI. Cramer's *v* was used to assess the strength of association, with *d* = 0.20–0.50 considered a small effect, *d* = 0.50–0.80 medium, and *d* ≥ 0.80 large (27). A point-biserial correlation was used to explore whether age was associated with providers’ follow-up recommendations.

The R Statistical Environment (28) and RStudio Integrated Development Environment (29) were used for all cleaning and analysis procedures. The tidyverse (30) package was used for data cleaning and preparation, and the psych (31), stats (28), rstatix (32), pwr (33), and effectsize (34) packages were used for assumption checks, power analysis, and data analysis.

## 3 Results

### 3.1 Demographics

A description of the demographic and injury-related characteristics for the sample of *n* = 50 patients with PNI is presented in Table 1. The majority of participants were male, with an average age of 45.2 years (*SD* = 16.9, range 19–79). Traumatic injuries were the most common etiology, specifically motorcycle crashes and motor vehicle accidents. Most participants were right-hand dominant, and in the majority of cases, the dominant hand was unaffected by the PNI.

**Table 1:** Demographic and injury characteristics.

Characteristic	Total, *N* = 50 [*n* (%) or *M* (SD)]	Surgical Patients, *N* = 32 [*n* (%) or *M* (SD)]	Non-Surgical Patients *n* = 18
Age	45.2 (16.9)	45.9 (15.6)	44.7 (18.6)
Sex			
Male	32 (64)	19 (59.4)	13 (72.2)
Female	18 (36)	13 (40.6)	5 (27.8)
Hand Dominance			
Right	48 (96)	30 (93.8)	18 (100.0)
Left	0 (0)	0 (0)	0 (0)
Both/Unknown	2 (4)	2 (6.3)	0 (0)
Injury Laterality			
Right	22 (44)	15 (46.9)	7 (38.9)
Left	26 (52)	15 (46.9)	11 (61.1)
Both	2 (4)	2 (6.3)	0 (0)
Dominant hand injured			
Yes	24 (48)	17 (53.1)	7 (38.9)
No	26 (52)	15 (46.9)	11 (61.1)
Mechanism of Injury			
Traumatic	21 (42)	13 (40.6)	8 (44.4)
Iatrogenic	12 (24)	9 (28.1)	3 (16.7)
Other/Chronic	17 (34)	10 (31.3)	7 (38.9)

M, mean; SD, standard deviation.

### 3.2 Patient perceptions

**Figure 1:**
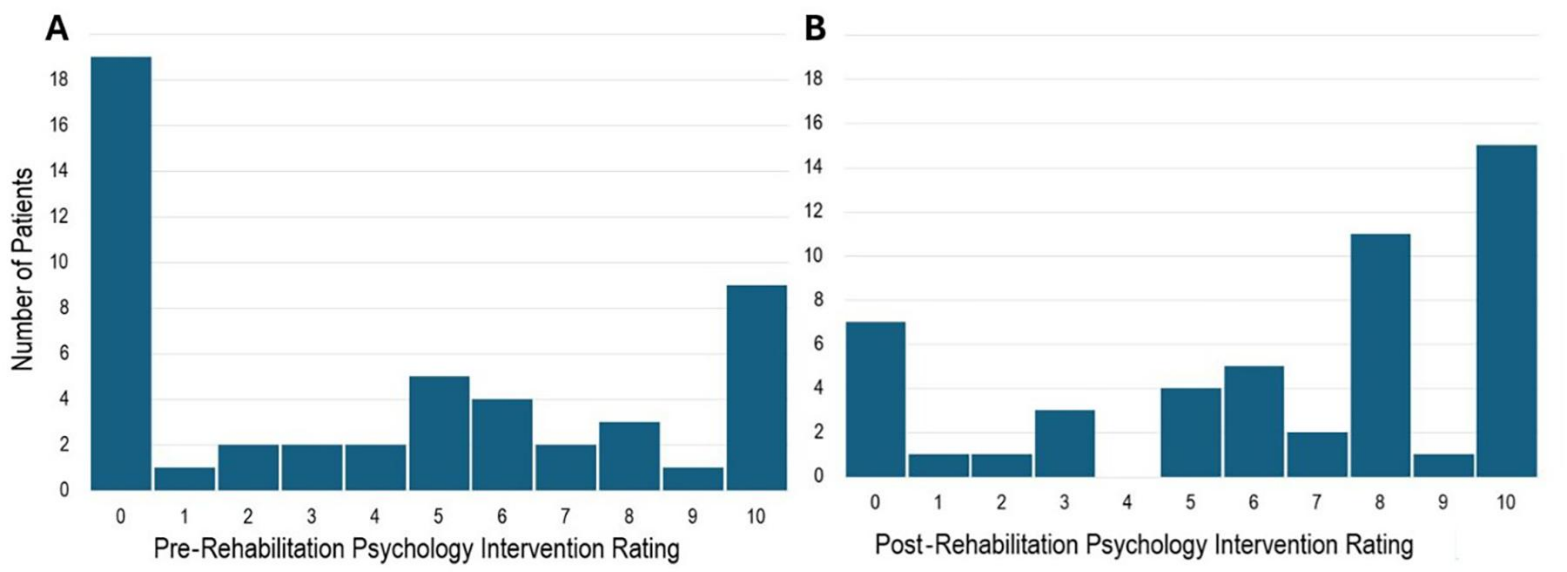
Frequency distributions of participant ratings for **(A)** Pre-rehabilitation psychology session and **(B)** post-rehabilitation psychology session. The data illustrates a rightward shift in ratings following the intervention, indicating improved patient ratings and perceptions of rehabilitation psychology. Ratings ranged from a minimum score of 0 to a maximum score of 10.

**Figure 2:**
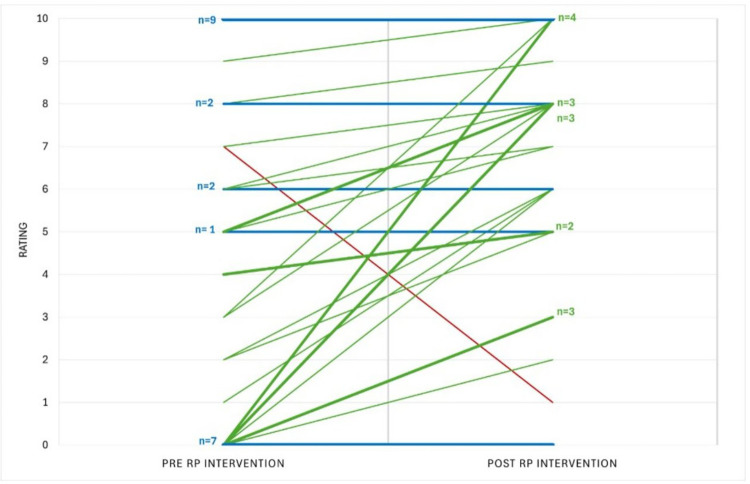
Individual participant ratings before and after rehabilitation psychology intervention. This figure displays participant ratings for all fifty participants before (Pre-RP Intervention) and after (Post-RP Intervention) the rehabilitation psychology intervention. Each green line represents a participant with a positive change in score; when multiple participants had the same change of score, the corresponding number is shown to the right of the line. Each blue line represents no change in score, with the number of participants denoted on the left side of each line. The single red line represents the one participant who had a decreased change in score. Ratings ranged from 0 (lowest) to 10 (highest).

The distribution of pre- and post- consultation ratings demonstrated a rightward shift, with substantially more patients providing higher scores post-consultation (Figure 1). Most participants (56%) reported an increase in perceived value following the RP session, 42% reported no change, and one participant (2%) reported a decrease (Figure 2). Overall, 74% of participants either showed improvement or maintained a maximum pre- and post-visit score of 10, indicating an overall increase in perceived usefulness of the RP service.

Results from Levene's tests suggested that the homogeneity of variance assumption was not violated for any of gender, injury etiology, mechanism of injury, operative status, and if patients’ dominant hand was affected (*p* = 0.16–0.97), so equal variances were assumed for all independent t-tests and for the ANOVA. However, while the skew (0.8) and kurtosis (0.26) for the change scores of patients’ perceptions of RP were both within bounds of the established criteria, the Shapiro–Wilk test was statistically significant, *w* = 0.83, *p* < .001.

We used the Wilcoxon signed-rank test in place of the paired samples *t*-test and used the rank-biserial correlation (*r*) for the effect size. Results suggested that there was a significant and large increase in patients’ reported perception of the value of RP services from before meeting with the rehabilitation psychologist (*Mdn* = 4, IQR = 7.75) to after (*Mdn* = 8, IQR = 5.00), *z* = 414.50, *p* < .001, *r* = 0.68.               

Mann–Whitney U tests were used in place of independent *t*-tests and again used the rank-biserial correlation for the effect size. Results suggested there was a significant and small effect for gender, such that men (*Mdn* = 2) experienced greater improvement in perceived value compared to women (*Mdn* = 0), *W* = 193, *p* = .047, *r* = 0.28. Change in the perceived value of RP did not significantly differ based on operative status, though a small effect was present suggesting that nonoperative patients (*Mdn* = 2) may have experienced greater change compared to nonoperative patients (*Mdn* = 0.5), *W* = 228, *p* = .436, *r* = 0.12. Perception change did not significantly differ based on whether patients’ dominant hand was affected by their PNI, *W* = 314, *p* = .952, *r* = 0.01.

We conducted Kruskal–Wallis tests in place of one-way ANOVAs and used *η*^2^ for the effect size. Results indicated that patients’ change in perception did not differ across mechanism of injury, *χ*^2^(8) = 2.29, *p* = .971, *η*^2^ = 0.14, nor across injury etiology, *χ*^2^(3) = 0.21, *p* = .977, *η*^2^ = 0.06. While moderate and small effects were present, respectively,       we elected not to investigate these further given the number of levels for each variable, as this resulted in too small of a sample in each level to produce any interpretable results.

Finally, we used a Spearman correlation in place of the simple linear regression. This test suggested that age was not significantly related to patients’ change in perception, *ρ* = −0.09, *p* = .538.      

### 3.3 Provider perceptions

**Figure 3:**
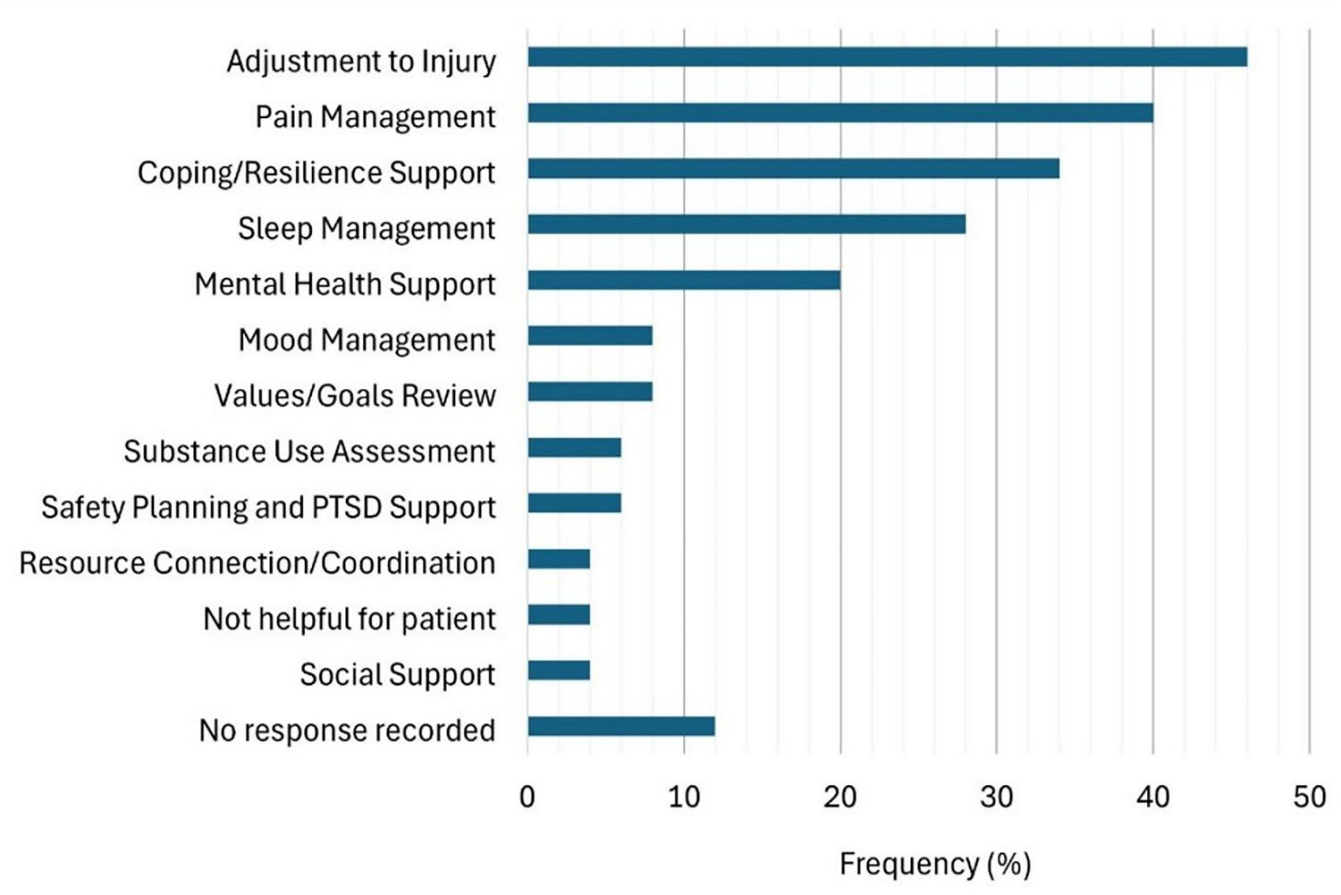
Topics addressed during rehabilitation psychology sessions. This bar chart illustrates the frequency (in percentage) of primary focus areas addressed during rehabilitation psychology sessions, as reported in rehabilitation psychologist provider surveys.

Provider data was available for 44 of the 50 patients included in the present study. Of these 44 patients, providers recommended that 15 (34.1%) would benefit from follow-up with RP services. The most frequent themes from provider recommendations and topics of discussion during RP sessions were Adjustment to Injury (46%), Pain Management (40%), and Coping and Resiliency Support (38%) (Figure 3). Overall, chi-square analyses showed that provider recommendations did not have any relation with gender (*χ*^2^ = 2.56, *df* = 1, *p* = .109, *v* = 0.25), mechanism of injury (*χ*^2^ = 8.40, *df* = 8, *p* = .396, *v* = 0.07), operative status (*χ*^2^ = 1.29, *df* = 1, *p* = .256, *v* = 0.18), or whether the dominant hand was affected (*χ*^2^ = 0.40, *df* = 1, *p* = .525, *v* = 0.00). Although these relations did not reach statistical significance, there was a small effect size present for gender that suggested a potential association that may not have been detectable given the limited sample size. To further explore this pattern, a binary logistic regression model was used to calculate an odds ratio, which indicated that providers were more likely to recommend women to follow-up with rehabilitation psychology services than for men (OR = 3.57). Participants’ age was also not significantly associated to provider recommendations (*r* = −0.18, *p* = .230).

## 4 Discussion

This study explores the perceived impact of integrating RP services into a multidisciplinary clinic for patients with upper extremity PNIs. Overall, our findings suggest that psychological support is perceived as beneficial across the PNI patient population, as reflected in consistently high or increased positive ratings of perceptions of RP following intervention. The only significant demographic predictor of increased perceived benefit was male sex. Despite this difference, the overall positive response across participants supports offering RP services broadly to all PNI patients, rather than targeting specific subgroups. As such, these findings support the integration of RP services as a core component of multidisciplinary PNI care pathways across all populations, highlighting the of psychological support as an essential element of comprehensive PNI management rather than a selective adjunct service.

Importantly, 74% of patients either reported an increase in their positive perception of RP or maintained the highest possible score before and after the session, indicating that the vast majority found psychological support valuable. This finding supports the clinic's approach of offering RP consultations to all new patients as part of standard care in the multidisciplinary clinic. Notably, male patients demonstrated significantly greater increases in perceived benefit, which may suggest lower initial expectations or greater skepticism toward psychological services compared to female patients, thus allowing more room for positive change following intervention (35). Even more broadly, patients who had initially rated RP as having low importance but later reported an increase in perceived value following their intervention exemplify the impact even a single session can have. Consistent with prior literature that even brief psychological interventions can reduce distress and improve adjustment (36, 37), our study similarly shows how a single consultation helped patients gain a clearer understanding of how pain psychology, emotional support, and coping strategies can meaningfully support their recovery. These shifts in perception illustrate how even patients who are unfamiliar with psychological services, or uncertain of their relevance, can come to appreciate the benefits of a rehabilitation psychologist's role in their recovery (38). This pattern may possibly reflect a general lack of prior knowledge or exposure to psychological services, especially in patients who may not consider themselves as having mental health concerns. It also highlights the potential for even brief interventions to shift patient thoughts about recovery and to reframe psychological care from being problem-focused to being proactive and supportive.

In contrast, the sub-cohort of “zero change” responses represent a different but equally meaningful form of impact. of these patients (*n* = 21) consistently reported high ratings (eg., “10 to 10”) from pre- to post-intervention, suggesting a strong pre-existing appreciation for psychological support, possibly shaped by prior mental health experience, higher health literacy, or history of coping with chronic illness. From there, this may contribute to a greater openness to psychological support (36, 38). Others in the “zero change” sub cohort reported mid to high range values (“5 to 5” to “9 to 9”), indicating a generally positive view of mental health services but perhaps less perceived need for additional psychological support. Additionally, 7 patients reported zero change response from “0 to 0”, which may have reflected disengagement from or limited interest in psychological services. In the setting of a multidisciplinary approach, these RP interventions may help reinforce and validate patients’ existing values, meeting their expectations and affirming the role of psychological support in their recovery process. Furthermore, these findings support the importance of maintaining equitable access to RP services for all patients (39, 40). Even those who with strong preexisting understanding of psychological care and high levels of motivation for recovery may still meaningfully benefit from structured psychological support through a single RP session in this multidisciplinary outpatient setting.

From the provider perspective, the role of RP intervention is focused on managing common challenges faced by patients with peripheral nerve injuries, including coping with chronic pain, adjusting to functional limitations, and preparing for surgery or rehabilitative therapies. These sessions often address issues such as pain catastrophizing, anxiety about long-term function, and distress from disruptions in role identity (40). By integrating RP into a multidisciplinary clinic setting, this allows patients to have a dedicated space to process their injury and recovery experiences, to voice concerns that may not come up in a traditional surgical consultation, and to start developing mental health c mechanisms to support their recovery. Furthermore, rehabilitation psychologists also serve as a triage role to screen and assess the severity of psychological distress related to injury and recovery, as well as to determine which patients would benefit from longitudinal psychological support. This may include referring them to appropriate outpatient mental health services, pain psychology, or trauma-focused therapy. Early identification and intervention can make a real difference in ensuring that patients get the necessary and appropriate psychological care.

This pilot study has several limitations. First, this study was exploratory and used multiple statistical tests, resulting in an increased familywise Type I error risk. Statistical significance should therefore be interpreted with caution, with greater emphasis placed on effect sizes in this study. Further, since this study was conducted at a single institution, our results may not be generalizable to other clinical settings with different resources, structures, or patient populations. Even despite our clinic's wide geographic range and diverse patient populations, the outcomes may still be influenced by institution-specific practices. Additionally, although patient-reported data was collected prospectively with no restrictive exclusion criteria in an effort to reduce selection bias and improve inclusivity, this approach may introduce clinical heterogeneity that affects outcome interpretation. Furthermore, the small sample size, the absence of a control group, and the observational design create limitations on the ability to draw causal inferences. The sample population may also have been subject to selection bias from participant self-selection, as patients with negative views toward RP may have declined to enroll in the study. Finally, the patient survey instrument was brief (limiting detail) unvalidated, and the questions for the rehabilitation psychologist were about the clinic broadly and therefore needed to be interpreted about each specific patient, thereby providing limitations to applicability and generalizability. Despite these limitations, this preliminary work has provided interesting and valuable insights into patient perceptions of RP services, and highlights opportunities for future research to explore their impact on patient engagement, recovery outcomes, and multidisciplinary care integration.

Future research should incorporate validated measurements and longitudinal assessments to better evaluate the lasting impact of RP on patient outcomes. Tools such as the Patient Health Questionnaire-9 (PHQ-9) (41) or the World Health Organization-5 (WHO-5) Well-Being Index (42) may help track changes in psychological distress, emotional wellbeing, and quality of life over time. It would also be valuable to explore factors such as self-efficacy or readiness for change, which may influence patient engagement or outcomes. Furthermore, expanding the study population beyond upper extremity injuries and across multicenter sites would help improve the generalizability of RP integration across diverse patient populations. Additionally, future studies should consider incorporating qualitative methodologies, such as interviews or focus groups. Finally, in order for RP interventions to be integrated as a standard in multidisciplinary care, research should be conducted to investigate implementation barriers. These may include time constraints, staffing limitations, and reimbursement challenges, all of which may affect the scalability and sustainability of RP integration. Early studies like this can help identify which patients are most likely to benefit and when in their treatment trajectory RP interventions are most effective. This insight is key to developing care pathways that are both patient-centered and resource-efficient.

## 5 Conclusion

RP intervention integrated within a multidisciplinary peripheral nerve clinic may have potential benefits in improving patient perception of their injury and treatment process. While no statistically significant associations were found between perception changes and clinical factors, many patients appeared to find value in a single RP session. With a notable majority of 74% of participants who demonstrated an increased belief in the value of RP following the session or reported maximum belief in value that remained constant from pre- to post-session, this supports the value of including RP in this multidisciplinary clinical setting. Further research should explore the long-term role of RP and assess applications beyond upper extremity peripheral nerve injuries.

## Data Availability

The datasets will be made available from the corresponding author upon reasonable request.
